# Iliac Crest Bone Block Autograft Transfer for Ballistic Posterior Glenoid Fracture: A Case Report

**DOI:** 10.1155/cro/5565275

**Published:** 2025-01-06

**Authors:** Jordan Cook Serotte, Hayden Baker, Cody Lee, Jason A. Strelzow

**Affiliations:** ^1^Department of Orthopaedic Surgery, University of Chicago Medical Center, Chicago, Illinois, USA; ^2^Department of Orthopaedic Surgery, Washington University School of Medicine in St. Louis, The University of Chicago, St. Louis, Missouri, USA

## Abstract

**Case:** A 25-year-old male presented with a ballistic fracture of the right glenoid resulting in > 30% loss of the posterior glenoid articular surface and acute posterior glenohumeral instability that was treated with open reduction internal fixation with iliac crest autograft transfer.

**Conclusion:** There is limited consensus on the operative management of ballistic intra-articular fractures due to the heterogeneity of these injuries. Acute posterior glenohumeral instability secondary to a ballistic fracture is a rare injury pattern. In this case, we were able to successfully treat posterior glenohumeral instability with iliac crest autograft transfer and open reduction internal fixation.

## 1. Introduction

The majority of urban ballistic fractures result from lower energy projectiles [1, 2]. Ballistic injury patterns result from strong deforming forces that are often accompanied by significant damage to the underlying soft tissue from the blast effect. Limited literature exists on acute operative management principles for these injuries.

Posterior glenohumeral instability is a less common cause of shoulder instability, accounting for 2%–10% of all cases [3]. This encompasses a large spectrum of injury, ranging from subtle posterior subluxation to frank posterior dislocation. The most common etiology of posterior instability is cumulative microtrauma leading to attenuation of the posterior labrum and capsule [3, 4]. Acute traumatic injuries resulting in posterior glenoid fracture dislocations are less commonly reported. They represent 0.9% of shoulder fracture–dislocations and have an annual incidence of 0.6/100,000 [5, 6]. High energy trauma (67%), seizures (31%), and electrocution (2%) are the most common etiologies of acute posterior glenoid fracture dislocations [7–9].

The literature reports good clinical outcomes after arthroscopic posterior stabilization, with low recurrence rates and high patient satisfaction. Similar to anterior glenoid bone loss, the amount of posterior glenoid bone loss has been shown to be linearly associated with higher failure rates with a posterior capsulolabral repair [10].

Whether performed open or arthroscopically, a variety of reconstructive procedures have been described for cases of glenohumeral instability with clinically significant posterior glenoid bone loss [11–13]. Posterior bone block transfer may include iliac crest autograft or distal tibial allograft transfer. Additional surgical treatment may consist of rotational osteotomy, glenoid osteotomy, or arthroplasty [4, 10, 11]. A recent systematic review of posterior shoulder fracture dislocations identified 148 cases published in the literature from 2007 to 2016 [8]. There were no reported cases of ballistic injuries in this review [8]. The most common fixation strategy in the review was open reduction internal fixation, followed by allograft and autograft reconstruction [8]. We report a case of a ballistic posterior glenoid fracture–dislocation treated with open reduction internal fixation and iliac crest bone block autograft transfer.

## 2. Case Report

**Figure 1:**
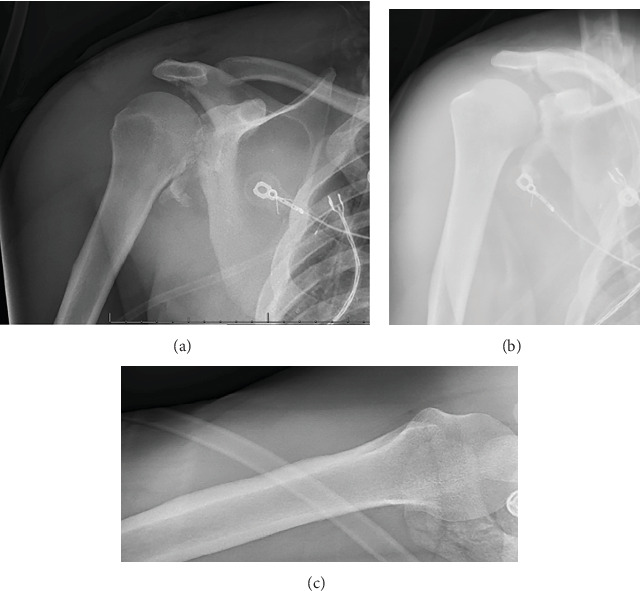
(a) AP XR of the right shoulder. (b) Grashey XR of the right shoulder. (c) Axillary XR of the right shoulder.

**Figure 2:**
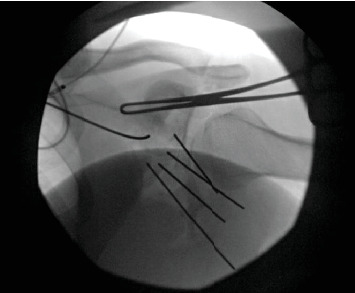
Intraop XR of the right shoulder.

**Figure 3:**
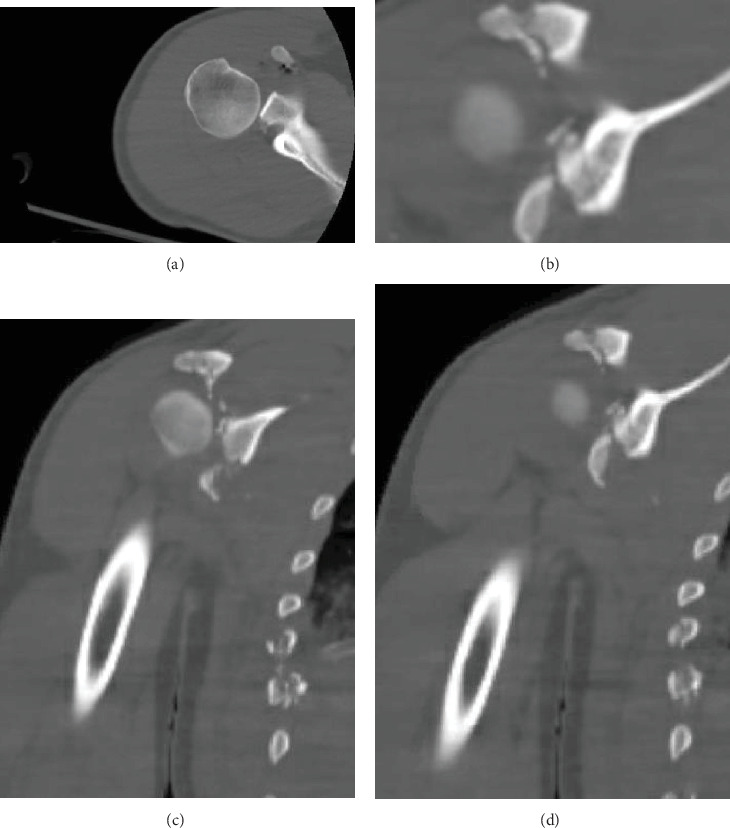
(a) Axial CT of the right shoulder. (b) Axial CT 2 of the right shoulder. (c) Coronal CT of the right shoulder. (d) Coronal CT 2 of the right shoulder.

**Figure 4:**
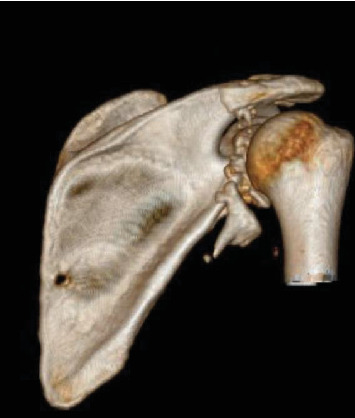
3D reconstruction of the right shoulder.

A 25-year-old man with no significant past medical history and a body mass index (BMI) of 21.5 kg/m^2^ presented to the emergency department with nine gunshot wounds. The patient underwent emergent exploratory laparotomy and multiple subsequent surgeries by the general surgery team. Orthopaedics was consulted for multiple ballistic fractures including the right acetabulum, acromion, and glenoid. Of note, his other injuries included ballistic T7 ASIA B spinal cord injury and bilateral hand fractures managed by neurosurgery and plastic surgery, respectively. On physical examination of the right upper extremity, the patient had a small, 1 cm ballistic wound over the superior shoulder with associated soft tissue swelling. He was neurovascularly intact distally in the right upper extremity with noted intact sensation over the lateral shoulder and the ability to actively fire his deltoid. Imaging showed a significantly comminuted posterior glenoid fracture with bony loss of > 30% of the articular surface, significant chondral damage, and posteroinferior subluxation of the humeral head. Images of the right shoulder are pictured in Figures 1, 2, 3, and 4. Given the significant bone loss leading to acute posterior glenohumeral instability and his associated lower extremity paraplegia, we recommended the patient undergo open reduction internal fixation of the glenoid with iliac crest bone autograft transfer. Three weeks after the injury, the patient was cleared by the intensive care unit for shoulder surgery.

**Figure 5:**
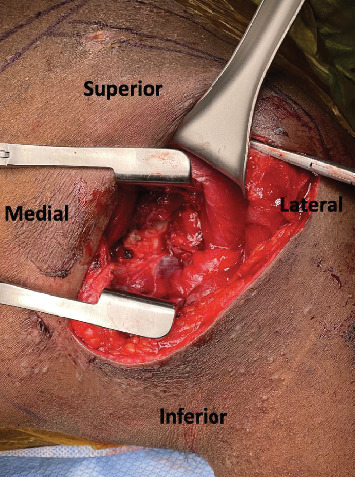
Intraoperative view of the posterior glenoid.

Under general anesthesia, the patient was positioned prone, and we performed a reverse Eden–Hybinette procedure (posterior glenoid reconstruction with iliac crest autograft transfer). We used a modified Judet approach, using only the vertical limb for the exposure. We exposed from the posterolateral lip of the acromion to the lateral border of the scapula and distally along the lateral border towards the inferior angle. We retracted the deltoid superiorly and developed the interval between infraspinatus and teres minor to mobilize infraspinatus and teres minor. We then performed a capsulotomy to expose the joint. We promptly encountered bony debris and free-floating articular cartilage (Figure 5). The capsule and labrum were tagged for later repair. At this point, it was easy to visualize the dynamic posterior glenohumeral translation of the humeral head with gentle manipulation of the arm, and an almost 50% defect of the posteroinferior humeral head was noted.

Given the severity of comminution, we felt that no degree of native bony reconstitution was feasible. We, therefore, planned a reverse Eden–Hybinette using a harvested bone graft from the posterior iliac crest. We made a 5-cm incision over the posterior crest and exposed a 12 × 7 mm block of bone to serve as our bone graft using standard technique.

**Figure 6:**
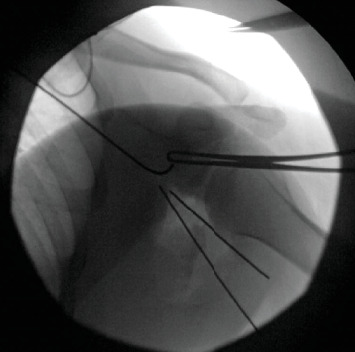
XR preliminary fixation of the glenoid bone graft.

**Figure 7:**
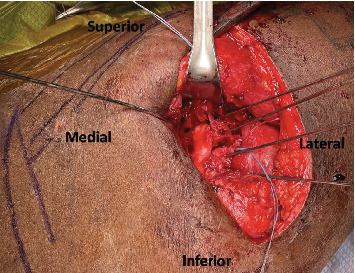
Intraoperative view of preliminary fixation of the glenoid bone graft.

**Figure 8:**
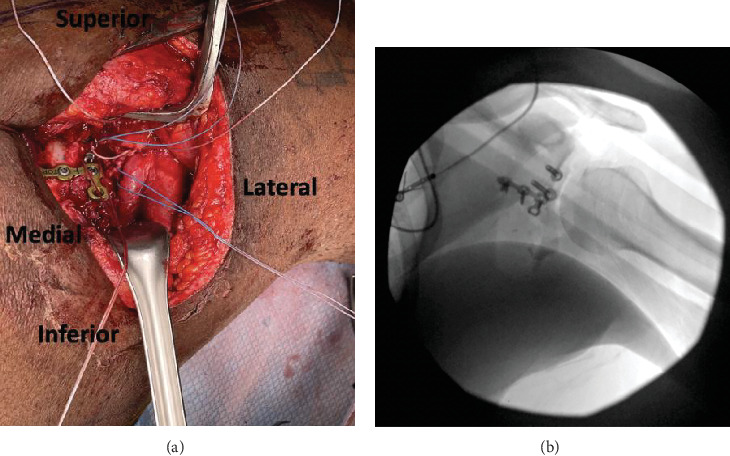
(a) Intraoperative view of T-plate over the posterior glenoid bone graft. (b) XR T-plate over the posterior glenoid bone graft.

To affix the graft, we used a commercial glenoid bone loss system. Two 4-mm pilot holes were drilled through the graft to ensure that the cancellous raw, bony surface would be facing the articular margin of the fractured glenoid. The graft was then loaded onto the offset jig, and we placed our bone graft flush with the fracture. Next, we placed two guidewires to preliminarily fixate the graft while we checked to ensure the position of the graft was acceptable (Figures 6 and 7). Radiographs confirmed a concentrically reduced shoulder with restoration of the articular surface and good positioning of our bone block. In a lag-by-technique fashion, we placed 37-mm fully threaded screws with suture washers. Next, we placed a 2.7-mm locking T-plate over the lateral inferior margin of the scapula (Figure 8). Given the significant comminution of the glenoid, we believed further fixation with a T-plate was necessary. We then evaluated the nonarticular fragmentation that remained and was indeed neither intra-articular nor likely to be a source of impingement. Removal would have required additional exposure and further potential trauma to the articular surface. Therefore, this fragmentation was treated nonoperatively. Finally, the suture washers were used to secure the posterior labrum and capsule to the rim of the articular surface, which was able to fully encapsulate the bone block.

Final imaging confirmed a concentric reduction of the joint. Radiographically, given the lack of cartilage on the bony fragment, the graft did not appear recessed. The medial and lateral columns were restored with an articular reduction. Wound closure was performed with Maxon in fascia followed by skin closure. The patient was made nonweight bearing to the right upper extremity with range of motion (ROM) as tolerated postoperatively.

**Figure 9:**
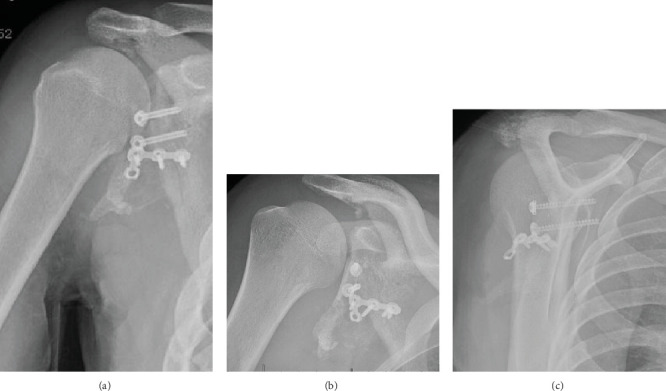
(a) AP right shoulder 1 month postoperatively. (b) Grashey XR right shoulder 1 month postoperatively. (c) Scapular Y XR right shoulder 1 month postoperatively.

**Figure 10:**
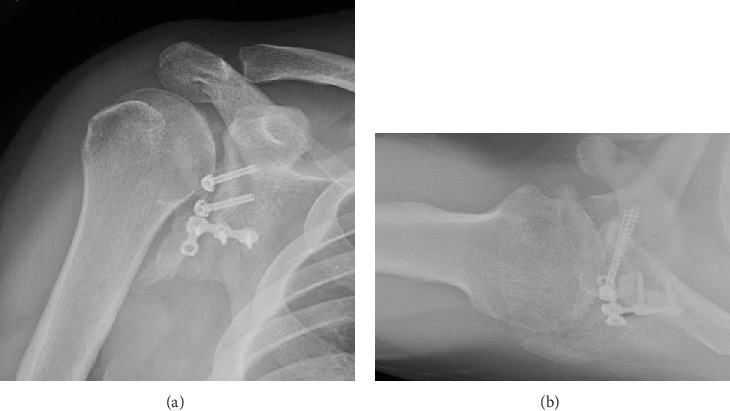
(a) AP XR right shoulder 6 months postoperatively. (b) Axillary XR right shoulder 6 months postoperatively.

At the 1-month postoperative visit, the patient was neurovascularly intact on the operative extremity but had limited active ROM (Figure 9). He was referred to physical therapy. ROM at subsequent postoperative visits (6 and 18 months) is shown in Table 1. Imaging from his postoperative appointment at 6 months is shown in Figure 10. There is a small step-off noted at the graft-glenoid interface with slight absorption and interval development of degenerative changes. We suspect that some of these degenerative changes are secondary to the ballistic osteochondral injury to the humeral head. At the final follow-up, 18 months postoperatively, his disabilities of the arm, shoulder, and hand (DASH) score was 52.3. His T7 paraplegia remained without recovery of lower limb function. The patient reported difficulty with heavy household chores, carrying a shopping bag/briefcase, washing his back, and did not return to work since his injury. However, he related his difficulties with performing the above tasks to his lower extremity paraplegia. He reported intermittent right shoulder pain. At his latest visit, he saw an advanced practicing nurse who tested ROM as well as an outcome score, with no imaging taken at this appointment.

## 3. Discussion

This case study reports a relatively rare case of posterior glenohumeral instability secondary to a ballistic fracture with approximately 50% glenoid bone defect. Currently, there are no guidelines for which posterior glenoid bony augmentation is definitively recommended despite hundreds of articles published on the importance of treating anterior glenoid bone loss and instability. Literature has shown that young patients with a displaced posterior glenoid fracture and posterior instability should be fixed with open reduction internal fixation [8]. Extrapolating from the literature on anterior glenoid bone loss, patients with posterior glenoid bone loss greater than 20% of the glenoid surface area may benefit from bony augmentation. However, there are several factors to consider that are specific to posterior glenoid bone loss, including excessive glenoid retroversion or hypoplasia, and bipolar bone loss. Recently, Arner et al. published a case series that demonstrated posterior glenoid bone loss of 11%, which is associated with a 10.4 times higher failure rate of soft tissue surgical repair [10]. In our case, the amount of posterior glenoid bone loss (~50%) far exceeded any previously proposed thresholds for bony augmentation. We believe this is the first reported case of bony augmentation of the posterior glenoid performed for a ballistic fracture.

In our case, given the severity of comminution and acute traumatic setting, we chose to perform open bony augmentation using a posterior iliac crest bone graft. Previous cadaveric laboratory studies have shown similar biomechanical properties including peak force, contact pressure, and contact area when comparing posterior glenoid augmentation with iliac crest autograft and distal tibia allograft [14]. Most of the literature for glenoid bone grafting is in the context of a reverse total shoulder arthroplasty when the graft is used for structural support in a deficient glenoid. The authors prefer to use allograft/autograft from the humeral head if the patient's bone quality allows for it. In our case, we chose to use iliac crest autograft over distal tibial allograft because the patient was admitted to the ICU with bacteremia secondary to his subdiaphragmatic loculated infection. Therefore, we believed he was a better candidate for autograft given his recent bacteremia.

Recently, Mojica et al. published a systematic review evaluating the outcomes of bone block augmentation in the treatment of posterior glenohumeral instability. The authors found good patient-reported outcome scores measured by Rowe and Walch–Duplay (range 75–89 and 76–90, respectively [average scores of 81.4 and 81.6, respectively]). Although 11.6% of patients reported residual shoulder pain at final follow-up, recurrent instability was 9.78%, including 7 re-dislocations, 3 subluxations, and 2 apprehension events [11]. We speculate that despite the significant glenoid bone loss in our case, the nature of acute penetrating trauma limited the cumulative microtrauma typically seen in conventional posterior glenoid instability. We believe this contributed to our patient's overall continued stability postoperatively. Our case suggests that posterior glenoid bony augmentation is a viable procedure for patients with ballistic fractures of the glenoid with significant bone loss. Further studies on the degree of posterior glenoid bone loss that warrants bone augmentation in the setting of ballistic fracture are warranted.

## Figures and Tables

**Table 1:** ROM at 6 and 18 months postoperatively.

**Range of motion**	**6 months postoperatively**	**18 months postoperatively**
Forward flexion	110°	120°
Abduction	90°	100°
External rotation	Not recorded	40°
Internal rotation	Not recorded	L3 level

## Data Availability

The data that support the findings of this study are available from the corresponding author, J.C.S., upon reasonable request.
