# Corrigendum to “Iliac Crest Bone Block Autograft Transfer for Ballistic Posterior Glenoid Fracture: A Case Report”

**DOI:** 10.1155/cro/9790302

**Published:** 2025-06-05

J. C. Serotte, H. Baker, C. Lee, and J. A. Strelzow, “Iliac Crest Bone Block Autograft Transfer for Ballistic Posterior Glenoid Fracture: A Case Report”, *Case Reports in Orthopedics* 2025, no. 1 (2025): 1–7, https://doi.org/10.1155/cro/5565275

In the article titled “Iliac Crest Bone Block Autograft Transfer for Ballistic Posterior Glenoid Fracture: A Case Report,” there was an error in Affiliation Number 2. In the corrected affiliation, “The University of Chicago” has been removed. The corrected affiliation appears below:

“Department of Orthopaedic Surgery, Washington University School of Medicine in St. Louis, St. Louis, Missouri, USA”

We apologize for this error.

